# The role of unsustainable HR practices as illegitimate tasks in escalating the sense of workplace ostracism

**DOI:** 10.3389/fpsyg.2022.904726

**Published:** 2022-09-23

**Authors:** Afaq Ahmad, Chenhui Zhao, Ghazanfar Ali, Kunshun Zhou, Jawad Iqbal

**Affiliations:** ^1^Institute of Business, Management and Administrative Sciences, The Islamia University of Bahawalpur, Bahawalpur, Pakistan; ^2^School of Business, Wuchang University of Technology, Wuhan, China; ^3^School of Business Administration, Zhongnan University of Economics and Law, Wuhan, China; ^4^School of Economics and Management, Guangxi Science and Technology Normal University, Liuzhou, China

**Keywords:** unsustainable HR practices, workplace incivility, illegitimate tasks, workplace ostracism, faculty members, private universities

## Abstract

Unsustainable HR practices impose illegitimate tasks on employees due to a shortage of resources. These illegitimate tasks bring counterproductive work behavior in terms of workplace incivility that creates a sense of ostracism in employees. To address these issues, the study examined the relationship among unsustainable HR practices in terms of illegitimate tasks and workplace ostracism. Whereas workplace incivility is defined as an underlying reason through which this association exists. Adopting a theoretical framework from earlier research, the study used cross-sectional data and subsequently a method of quantitative research, and the sample comprised permanent faculty members of private universities in Pakistan working in different departments with different role titles. Smart PLS was applied to run multiple statistics analyzed on the obtained data. The results from the study supported the hypothesis by depicting a positive and significant association between illegitimate tasks and workplace ostracism. Further, workplace incivility was playing the mediating role between illegitimate tasks and workplace ostracism. The results from the study carry significant implications for managers and researchers. Recommendations and future research directions are also discussed in the paper.

## Introduction

Managers and researchers in the field of human resource management (HRM) and organizational behavior are always keen on identifying such stressors that induce negative behaviors among employees. The importance of tasks in shaping workplace behaviors has gained the attention of researchers in recent years. Task-related stressors are important for an employee’s wellbeing and highly associated with the employee’s self in many ways. Many studies in the past have shed the light on these task-related stressors on several personnel-related factors (Fila and Eatough, 2020; Chen et al., 2021; Kilponen et al., 2021). Such stressors are associated with the number of employee-related outcomes in the workplace setting, yet research has not uncovered these outcomes and the path that leads to such outcomes in an academic institution.

Tasks will be considered illegitimate if the employees consider them to be not worthy of carrying out and unnecessary. The concept of illegitimate task is relatively new and considered as identity stressor that violates people’s professional individuality. Such task-related stressors can have negative consequences on employees as indicated by previous studies. Semmer et al. (2019) explained two reasons for which employees can perceive any task as illegitimate. They may see the task as unreasonable, which means they lie beyond the employee’s occupational role. Second, employees may consider the task as unnecessary, which means the work should have been avoided or not done at all. Such tasks are a threat to employees’ behavioral and work-related outcomes. Thus, an illegitimate task is considered by an employee for not carrying out and as a violation of what can be reasonably expected from them.

Many limitations in previous research studies have analyzed the negative outcomes of illegitimate tasks that need dire attention of researchers. Only, a limited number of outcomes have been discussed in previous studies as it is relatively a new term in the field of management and organizational behavior (Minei, 2022). Second, mixed results from previous studies pose an unclear picture of the role of illegitimate tasks that need to be addressed. Third, it is evident that illegitimate tasks are quite common in academia relative to the corporate sector, but previous studies are usually focused on corporate employees rather than faculty members. Furthermore, the theoretical perspective to analyze the illegitimate tasks in escalating negative outcomes is limited to the date.

In light of current literature on illegitimate tasks, several negative outcomes have been discussed by different studies such as occupational wellbeing, stress, employee expediency, employee performance, and role orientation (Ma and Peng, 2019; Chen et al., 2021; Minei, 2022). Researchers have emphasized the importance of understanding other negative outcomes associated with illegitimate tasks such as workplace ostracism. Workplace ostracism can be defined as the extent to which an employee feels ignored or excluded by other members of the organization (Minei et al., 2018). Scholarship on workplace ostracism discussed many antecedents that are associated with workplace ostracism, yet many other indicators have not been discussed in the available literature that requires the attention of researchers. Drawing on the theory of belongingness, when one perceives that injustice prevails in an organization and the top management is imposing extra work, such sense violates social norms and enhances deviant behavior like intention to leave the group (Baumeister and Leary, 1995; Thau et al., 2007). Further, when deviant behavior emerges, belongingness is thwarted (Pindek et al., 2019), resulting in adverse reactions such as rejection or ignoring from the group (Thun et al., 2018).

To fill the research gap and extend the present studies on illegitimate tasks, the present study utilizes belongingness theory to develop and examine the relationship of illegitimate tasks in escalating workplace ostracism in a way that workplace incivility is the underlying reason through which the illegitimate tasks are associated with ostracism at the workplace. In particular, we aim to address the following questions. How does the illegitimate task results in ostracism at the workplace? What is the possible mechanism through which the illegitimate tasks are associated with workplace ostracism? Are there any particular differences in the outcomes of illegitimate tasks in corporate and academia?

This study makes several contributions. First, several studies have investigated the negative outcomes of illegitimate tasks but this study is the first attempt to examine the association between illegitimate tasks and workplace ostracism. Second, the present study also contributed to the literature by examining the mediating role of workplace incivility in the proposed relationship. Third, this study utilizes the belongingness theory as a theoretical lens to understand the association among proposed variables, while the previous study often utilizes the theory of stress as an offense to self (Semmer et al., 2019). Furthermore, as indicated by previous studies, this study was conducted on faculty members of private universities as the frequency of illegitimate tasks in private universities is more frequent than in corporate sector and government universities. Finally, the results of the study have important implications for policymakers in higher education institutes as this can pose a serious threat to faculty members’ wellbeing. In a nutshell, the present theory provided a comprehensive theoretical view to advance the scholarship on illegitimate tasks and their negative consequences which contribute to the body of knowledge as well as help HEIs to take effective measures in de-escalating the illegitimate tasks to prevent negative outcomes for faculty members.

The present study follows an organized pattern to report all contents, starting with the introduction in the first section. The second section is about the hypotheses and theory development on illegitimate tasks, workplace incivility, and workplace ostracism in which we explore the literature for theory and variables to propose a hypothesis and theoretical framework. Next, section three will describe the research methodology including samples and techniques. Section four will present the empirical results from the gathered data. Section five will be based on the conclusions of the study, implications, and future research directions.

## Theory and hypotheses development

From the humanistic perspective, numerous practices regarding HRM are evolved to control employees’ lives over a period of time and it created harmful effects on workers (Mariappanadar, 2012). Further, the organization considers downsizing the employees to decrease payroll costs, however, paying no attention to the point that downsizing increases the workload of employees that effect negatively on the performance. So, these unsustainable practices refer to voluntary employee redundancy or turnover (Ehnert, 2009). There are numerous studies that propose that the unsustainable human resources management (UHRM) practices for applying to the workforce cause reduced productivity and employee turnover (Goetzel et al., 2003).

Mariappanadar (2014) explained that UHRM practices force the employees to get overworked just to sustain the high-performance work system. For example, the doctor often keeps on doing the work when he is ill, and due to the lack of staff, he has to perform extra-role tasks (ERTs) (Giæver et al., 2016; Thun and Løvseth, 2016). On the other hand, in the human resource literature, it is frequent to find that most UHRM practices employees perform because of a lack of resources, and tasks are assigned or imposed on employees who are in fact not responsible for performing them (Mariappanadar, 2013).

HR practitioners and researchers understand that the ERTs imposed by HR due to short of resources for profit maximization that refer to unsustainable practices (Mariappanadar, 2013). The lack of resources incites employees to perform administrative and operational tasks that fall beyond their role expectations that consider illegitimate tasks (Björk et al., 2013). These tasks are created through UHRM practices that provide harm to the employees, and employees can pay the price while performing these tasks (Mariappanadar, 2013). Further, the study by Björk et al. (2013) found that when an organization faces a shortage of resources, employees will bear to perform illegitimate tasks. The more the organization faces challenges in managing resources or incomprehensible decisional structure of the organization, the more the workload as illegitimate tasks are reported by the employees (Björk et al., 2013).

Likewise, the model of job demands–resources presented by Bakker and Demerouti (2017) makes clear how illegitimate tasks are due to the shortage of resources imposed by the managers to satisfy the demands (Semmer et al., 2015). So, it is mean when an organization wants to achieve a high target while having a lack of resource, and management starts imposing illegitimate tasks (beyond employee’s profession) or ERTs on their employees. Similarly, when supervisors impose ERTs in terms of workload on employees and these extra role tasks fall beyond the worker’s formal role, so, workers might perceive these tasks as a violation of their opportunities or role (Ohly et al., 2006). Further, role violations could be troublesome and generate psychological stress for the employees. Such employees might, consequently, assess the extra role tasks as unreasonable and, therefore, illegitimate (Björk et al., 2013). Faupel et al. (2016) conducted a study on faculty members and found that there were numerous tasks (such as supervision and extra duties) that were imposed on employees referred to as ERTs. These ERTs accounted as Illegitimate tasks (Semmer et al., 2010).

HR practitioners and researchers need to recognize that the excessive usage of human resources with internal competency to obtain the level of maximum profit leads to unsustainable HRM practices (Mariappanadar, 2013). Hence, managers must recognize that excessive working of such worthy employees (Ehnert, 2012) may produce counterproductive work behavior such as turnover intention and ostracism (Apostel et al., 2018). This study presents unsustainable HRM practices in terms of illegitimate tasks, because of short of resources that leads to workplace ostracism through workplace incivility. Moreover, performing an illegitimate extra task shuns to achieve performance objectives, since extra workload lessens energy and time that would be hindered for completing main tasks (Sonnentag and Lischetzke, 2018).

### Illegitimate tasks and workplace ostracism

Illegitimate tasks are a violation of norms by creating identity threats and low self-esteem (Semmer et al., 2007, 2015). According to belongingness theory, employee perceives low self-esteem and identity threat, while having work overload by the supervisor, and that perception leads to withdrawal behavior (Baumeister and Leary, 1995). If illegitimate tasks are performed by employees, then these tasks symbolize an important concern for mental health (Kottwitz et al., 2013), emotions (Turban and Cable, 2003), and strong withdrawal behaviors (Semmer et al., 2010) or disagreement (Goodboy, 2011).

*H1: Illegitimate tasks have a positive effect on workplace ostracism.*

### Illegitimate tasks and workplace incivility

Illegitimate tasks are a violation of norms that produce low self-esteem and identity threats (Semmer et al., 2007, 2015). According to belongingness theory, when an employee perceives injustice in terms of imposed workload, his self-esteem would be low and feels identity threat which leads to producing deviant behavior in the workplace (Baumeister and Leary, 1995; Thau et al., 2007). Further, Zhou et al. (2018) found that persons’ regular practices of illegitimate tasks may predict undesirable and deviant behavior. Thus, illegitimate tasks are the sources to pave the way to enhance workplace incivility in terms of deviant behavior at workplace.

*H2: Illegitimate tasks have a positive effect on workplace incivility.*

### Workplace incivility and workplace ostracism

Normally, workplace ostracism was studied in terms of ill-treatment constructs and was known as different constructs from other mistreatment constructs in the workplace (Robinson et al., 2013). Numerous mistreatment constructs such as sexual harassment, incivility, and workplace bullying as negative behavior toward a recipient, while WO includes the withdrawal behavior of the target employees (Ferris et al., 2008). However, workplace ostracism has a different effect on the individual compared to other mistreatment constructs. For example, workplace ostracism, compared to workplace bullying, has an effect on turnover intention, psychological withdrawal, and affective commitment (O’Reilly et al., 2015). Additionally, workplace ostracism has a greater effect compared to incivility and sexual harassment on professional efficacy, emotional exhaustion, and cynicism (Sulea et al., 2012).

Settles and O’Connor (2014) tested the practices of incivility and discourteous behavior—not only at the workplace but also at another atmospheres such as academic conferences, networking, and professional exposure. Women report more incivility, exclusion, and sexual harassment at conferences compared to men. Further, women faculty members report verbal aggression, gender harassment, exclusion, disrespectful behavior than men faculty members (Richman et al., 1999). These personal interactions have harmful outcomes as a turnover intention for women faculty (Cortina et al., 2013). It clearly demonstrates that the workplace incivility from others’ behavior compel women to create the perception of ostracism. Further, according to the belongingness theory, deviant behavior shuns a sense of belongingness (lower than desired) (Thau et al., 2007), and this sense realizes rejection or being ignored from the group (Baumeister et al., 1996).

*H3: Workplace incivility has positive effect on workplace ostracism.*

*H4: The mediating effect of workplace incivility between illegitimate tasks and workplace ostracism.*

### Methodology

The major goal of this study is to determine the outcomes of unsustainable human resource practices at private sector universities in Pakistan. Universities are independent and follow criteria issued by the higher education commissions. Because this study is quantitative as well as cross-sectional, data collection is confined to universities in Pakistan. Employees experience the burden of unsustainable HR practices at multiple levels inside institutions, and the notion of unsustainable HR practices is a prevalent problem. Questionnaires were utilized to gather data and were given to university faculty members other than the principal, chainmen, deans, and registrar, as well as Vice-Chancellor. It is because personnel at lower hierarchical levels consider unsustainable HR procedures. The participants came from several private university sector institutions. In all, 300 questionnaires have been distributed to respondents *via* convenience sampling as the population was unknown, and 208 were returned as suitable for data analysis, yielding a response rate of 69%.

### Measurement of variables

Here, the study has adopted the questionnaire from previous studies. It was based on a 5-point Likert scale. Illegitimate tasks from the Bern Illegitimate Tasks Scale have eight items to be measured (Semmer et al., 2010). That scale has two dimensions: (a) unnecessary tasks and unreasonable tasks. Ferris et al. (2008) developed workplace ostracism scale of 10 items, and further, 7 items of workplace incivility was developed by Cortina et al. (2001). The objective of the research requires researchers to proceed with quantitative analysis (i.e., deductive reasoning).

### Data analysis

**FIGURE 1:**
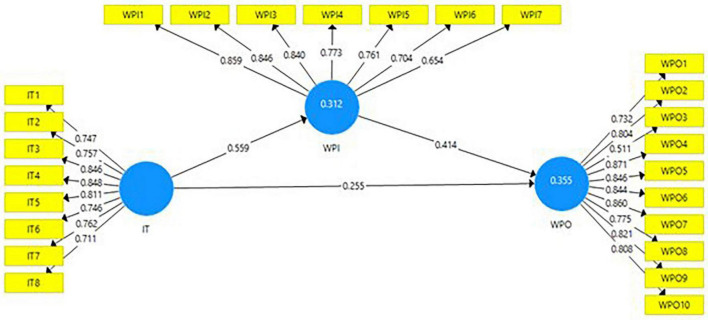
Model results.

Table 1 exhibited the CR for all the latent variables such as dependent, independent, and mediating variables, and the CR ranging from 0.704 to 0.871 showed a satisfactory level for all the constructs as internal consistency (Hair et al., 2012). In addition, it has also been found the AVE values lie between 0.608 and 0.630 and above 0.50 (Chin, 1998). The model results are shown in Figure 1.

**TABLE 1:** Outer loadings, composite reliability (CR), and average variance extracted (AVE).

Items	Factor loadings	Cronbach’s alpha	Composite reliability	Average variance extracted (AVE)
IT1	0.747			
IT2	0.757			
IT3	0.846			
IT4	0.848	0.907	0.925	0.608
IT5	0.811			
IT6	0.746			
IT7	0.762			
IT8	0.711			
WPI1	0.859			
WPI2	0.846			
WPI3	0.84			
WPI4	0.773	0.891	0.915	0.608
WPI5	0.761			
WPI6	0.704			
WPI7	0.654			
WPO1	0.732			
WPO2	0.804			
WPO3	0.511			
WPO4	0.871	0.932	0.944	0.63
WPO5	0.846			
WPO6	0.844			
WPO7	0.86			
WPO8	0.775			
WPO9	0.821			
WPO10	0.808			

### Discriminant validity

In contrast to CV, discriminant validity reflects the level at which a latent construct differs from others (Duarte and Raposo, 2010). However, likely to the CV, discriminant validity is calculated through AVE (Fornell and Larcker, 1981). The present study investigates the discriminant validity by using the method of Fornell and Larcker (1981). Table 2 exhibited the discriminant validity of the constructs. Further, the results display that the correlation values among all the latent variables are less than the square roots of AVE. Thus, it is confirmed that the constructs are distinct from other constructs and fulfill the criteria of discriminant validity.

**TABLE 2:** Discriminant validity.

Constructs	IT	WPI	WPO
IT	0.780		
WPI	0.559	0.780	
WPO	0.487	0.557	0.794

### Coefficient of determination (*R*^2^)

To evaluate the structural path model, the most important criterion to apply for the dependent variable is described as *R*^2^ determination (Hair et al., 2014b). The *R*^2^ number denotes how well the total variances of independent factors could be described by their dependent variable (Cohen, 1988). However, it is often assumed that the higher the *R*^2^-value, the greater the percentage of variations. As a result of the foregoing classification, and because this study used the PLS-SEM approach as its primary statistical analysis, *R*^2^-values are now more acceptable, as suggested by Chin et al. (1998). WPI has a value of 0.312 and WPO has a value of 0.335 of *R*^2^. Table 3 measures the R2 value of the constructs.

**TABLE 3:** Coefficient of determination (*R*^2^).

Constructs	*R* ^2^	*R*^2^ Adjusted
WPI	0.312	0.311
WPO	0.355	0.352

Measuring the association between unsustainable HRM practices (illegitimate tasks), workplace ostracism, and mediator workplace incivility, the process of PLS-SEM bootstrapping used 5,000 samples (Hair et al., 2014a). Table 4 exhibits the β-value, the *t*-values, *p*-values, and the decisions based on the results between exogenous variables and endogenous variables.

**TABLE 4:** Direct relationship.

Hypothesis	Relationship	Original sample (O)	Standard deviation (STDEV)	T Statistics (|O/STDEV|)	*P*-values	Decision
H1	IT - > WPI	0.559	0.054	10.279	0	Accepted
H2	IT - > WPO	0.255	0.062	4.103	0	Accepted
H3	WPI - > WPO	0.414	0.06	6.917	0	Accepted

All the results of direct hypotheses are shown in Table 4, and associations between illegitimate tasks and workplace incivility, illegitimate tasks and workplace ostracism, and workplace incivility and workplace ostracism are significant and positive and therefore all three hypotheses are accepted. In short, the positive significant association that are being accepted in the study includes, (i) IT- > WPI (β = 0.559, *p* = 0 < 0.05 and *t* = 10.279 > 1.96), (ii) IT - > WPO (β = 0.255, *p* = 0 < 0.05 and *t* = 4.103 > 1.96), and (iii) WPI - > WPO (β = 0.414, *p* = 0 < 0.05 and *t* = 6.917 > 1.96). These direct relationships are significant and accepted.

### Mediation

Following the structural path model, for bootstrapping process, this model is measured through PLS-SEM, 5,000 samples were used to measure the mediating effect of workplace incivility between illegitimate tasks and workplace ostracism. Hair et al. (2014a) found that bootstrapping is appropriate for mediation testing in PLS-SEM. By using PLS-SEM to measure the mediating effect that applied bootstrapping method of Baron and Kenny (1986).

This study examines the indirect relationship as shown in Table 5, the mediating effect of workplace incivility between illegitimate tasks and workplace ostracism. Further, IT - > WPI - > WPO (β = 0.232, *p* = 0 < 0.05 and *t* = 5.667 > 1.96). The mediating relationship is significant and accepted.

**TABLE 5:** Indirect relationship.

Hypothesis	Relationship	Original sample (O)	Standard deviation (STDEV)	T Statistics (|O/STDEV|)	*P*-values	Decision
H4	IT - > WPI - > WPO	0.232	0.041	5.667	0	Accepted

Scholars (Chin, 1998) suggested that Q^2^ for endogenous latent variables should be greater than zero (*Q*^2^ > 0). Therefore, the blindfolding procedure is conducted for cross-validated redundancy. In Table 6, the value of *Q*^2^ is greater than zero.

**TABLE 6:** Predictive relevance (*Q*^2^).

Constructs	SSO	SSE	Q^2^ (= 1−SSE/SSO)
IT	3,120.00	3,120.00	
WPI	2,730.00	2,254.62	0.174
WPO	3,900.00	3,097.77	0.206

## Discussion and conclusion

The current study is to examine the association of unsustainable HR practices in terms of illegitimate tasks on workplace ostracism including the mediating effect of workplace incivility. The first hypothesis (H1) examined the empirical investigation of illegitimate tasks and workplace incivility. The results showed that the β-value was found positive at 0.559 and t-statistics found higher than the cut-off point and observed as 10.279 > 1.96 with a *p*-value of 0.000, less than 0.05. Therefore, H1 was found to be positively significant. However, there is no finding of this relationship in previous studies. In private universities in Pakistan, faculty members have been imposed unnecessary and unreasonable tasks because of low resources by the management; this perception builds negative consequences in terms of incivility in the workplace. Faculty members get stressed and show deviant behavior in the workplace. The second hypothesis (H2) examined the empirical evidence of illegitimate tasks and workplace ostracism. The results showed that the β-value was found positive at 0.255 and t-statistics were found higher than the cut-off point and observed as 4.103 > 1.96 with a *p*-value of 0.000, less than 0.05. Therefore, H2 was found to be positively significant. The findings of this study are similar to the study of Apostel et al. (2018), who found that illegitimate tasks positively and significantly enhance turnover intention. In private universities of Pakistan, when faculty members have imposed these unnecessary and unreasonable tasks, they ultimately build the intention to leave the organization. They develop withdrawal behavior in the workplace.

The third hypothesis (H3) examined the empirical evidence of workplace incivility and workplace ostracism. The results showed that the β-value was found positive at 0.414 and t-statistics were found higher than the cut-off point and observed as 6.917 > 1.96 with a *p*-value of 0.000, less than 0.05. Therefore, H3 was found to be positively significant. There are numerous studies conducted on the comparison of workplace incivility and workplace ostracism (Ferris et al., 2017; Abubakar et al., 2018); these studies only found that workplace incivility is more harmful than workplace ostracism. However, in private universities of Pakistan, when faculty members face disrespectful behavior from the top management, they intend to leave that place. The fourth hypothesis (H4) examined the empirical evidence of mediating effect of workplace incivility between illegitimate tasks and workplace ostracism. The results showed that the β-value was found positive at 0.232 and t-statistics were found higher than the cut-off point and observed as 5.667 > 1.96 with a *p*-value of 0.000, less than 0.05. Therefore, H4 was found to be positively significant. There is no evidence of this mediating relationship. However, in private universities of Pakistan, when HR imposes unnecessary and unreasonable tasks, faculty members try to resist it. But they get disrespect and rude behavior from the HR management and faculty members will develop withdrawal behavior in the workplace.

### Implications

Employees’ unpleasant communications indicate a maltreatment spiral and the appearance of more malicious behaviors. However, managers must be energetically engaged and avert these work misbehaviors (Andersson and Pearson, 1999). Therefore, we suggest some valuable implications for the management of private universities of Pakistan. We recommend that leaders care about their employee’s responsibilities and job assignments and avoid those tasks that might seem illegitimate to them (Ali et al., 2018). Similarly, based on some elements such as professional skills and knowledge, tasks illegitimacy differs from person to person. Therefore, to reduce the level of the practices of illegitimate tasks, it is the obligation of the organization and its HR to recognize their worker’s skills as well as knowledge, and faculty members should be given responsibilities and tasks based on these abilities (Ali et al., 2018). We have faith in managers who modify their plan and support what employees want. So, our recommendation for experts is that they should make the system of recruitment, selection, training, and reward based on dealing with the perception of illegitimate tasks (Abubakar et al., 2018). Subsequently, to shield their employees from harmful consequences, we recommend HR management delegitimize such misbehaviors by enforcing hard rules against incivility (Zhao et al., 2022). Moreover, (Pearson and Porath, 2005) suggested that HR management should be responsible for appropriate training to teach faculty members how to deal with anxiety as well as conflict management, and avail career opportunities and friendly behaviors at work. Likewise, providing a resourceful atmosphere and making strong workplace harmony are worthwhile for employees. Such atmosphere may enhance employees’ workplace efficiency and sooner or later refers to positive organizational consequences (Zdaniuk and Bobocel, 2015). Thus, we recommend that the leaders should be responsible for assigning the right tasks for their staff and make an effort to avoid unnecessary and unreasonable tasks that might seem illegitimate to them which create counterproductive and withdrawal behavior in the minds of employees. The study focuses on employees’ perceptions about their responsibilities at workplace and its association between unsuitable HR practices and workplace ostracism. The negative perception of employees about unsustainable HR practices creates counterproductive work behavior.

### Limitations and future research course

The findings of the study should be understood regarding the study’s limitations. When the measurement was self-reported, it creates concern about common method variance (CMV) (Podsakoff et al., 2003). Further, the missing of a cross-lagged design of the study confined the causal conclusions which could be taken from the results. Moreover, the findings could not be generalizable through more effective resources to other national settings and the regulatory systems which could reduce workplace maltreatment. Fourth, the cultural perspective could have an important effect on findings. In future, researchers need to conduct similar studies from different cultural perspectives; longitudinal research may also give confirmatory evidence for the findings of the current study. Moreover, future studies may also identify factors that contribute to enhancing incivility in the workplace. Further, future research should examine the mediating variable between illegitimate tasks and workplace ostracism and the findings of the current study may direct such interventions. Psychological capital as a moderator may buffer the harmful effect of incivility.

## Data availability statement

The raw data supporting the conclusions of this article will be made available by the authors, without undue reservation.

## Author contributions

AA, CZ, and KZ contributed in the model development and analysis of the manuscript. GA and JI aided in data collection, drafting, and revision of manuscript. All authors contributed to the article and approved the submitted version.
